# Validation of a Brief Measure for Complicated Grief Specific to Reproductive Loss

**DOI:** 10.7759/cureus.37884

**Published:** 2023-04-20

**Authors:** Cara Buskmiller, Kathryn R Grauerholz, Jennifer Bute, Maria Brann, Michaelene Fredenburg, Jerrie S Refuerzo

**Affiliations:** 1 Obstetrics and Gynecology, Baylor College of Medicine, Houston, USA; 2 Hospital Medicine, Institute for Reproductive Grief Care, San Diego, USA; 3 Communication, Indiana University–Purdue University Indianapolis (IUPUI), Indianpolis, USA; 4 Communication Studies, Indiana University Purdue University Indianapolis, Indianapolis, USA; 5 Health Care Task Force, Institute for Reproductive Grief Care, San Diego, USA; 6 Obstetrics, Gynecology, and Reproductive Sciences, University of Texas Health Science Center at Houston McGovern Medical School, Houston, USA

**Keywords:** survey, abortion, stillbirth, miscarriage, neonatal death, postpartum depression, pregnancy loss, prolonged grief, screening, persistent complex bereavement disorder

## Abstract

Objective

Complicated grief reactions follow some pregnancy outcomes, like miscarriage, stillbirth, neonatal death, infant death, selective reduction, or termination of pregnancy. Stigma can delay treatment and worsen outcomes. Screening tools such as the Edinburgh Postnatal Depression Scale detect complicated grief poorly, and specific tools for prolonged or complicated grief after a reproductive loss are cumbersome. In this study, a five-item questionnaire to detect complicated grief after reproductive loss of any type was designed and preliminary validated.

Methods

A questionnaire patterned after the extensively validated Brief Grief Questionnaire (BGQ) was created by a group of physicians and lay advocates to employ non-traumatic but specific language related to grief after miscarriage, stillbirth, neonatal death, infant death, selective reduction, or termination of pregnancy. One hundred and forty women at a large academic center were recruited in person and via social media to validate the questionnaire with well-studied instruments for anxiety (7-item Panic Disorder Severity Scale, PDSS), trauma (22-item Impact of Events Scale), and reproductive grief and depressive symptoms (33-item Perinatal Grief Scale [PGS]).

Results

The response rate was 74.9%. Of the 140 participants, 18 (12.8%) experienced their loss during high-risk pregnancies, and 65 (46.4%) were recruited via social media. Seventy-one (51%) respondents had a score > 4, a positive screen for the BGQ. On average, women experienced their loss 2 years prior to participation (IQR 1-5 years). Cronbach's alpha was 0.77 (95% CI: 0.69-0.83). The goodness of fit indices of the model met Fornell and Larker criteria (RMSEA = 0.167, CFI = 0.89, SRMR = 0.06). The AVE was 0.42 and the CR 0.78.

Conclusions

This investigator-created screening tool is internally consistent and meets preliminary criteria for discriminant validity. This tool can be refined prior to testing for sensitivity and specificity in screening for complicated grief after a reproductive loss.

## Introduction

Every year, one to two million U.S. women experience reproductive loss, defined as the loss of a pregnancy or infant up to one year after delivery, whether spontaneous or associated with procedures such as selective reduction or termination of pregnancy [1-3]. Between 15% and 25% of women have symptoms of anxiety, depression, or post-traumatic stress after a reproductive loss [4-7]. Although these symptoms are classically pathognomonic for anxiety disorders, mood disorders, or post-traumatic stress disorder (PTSD), these same symptoms may in fact be due to the experience of loss. When symptoms like excessive worry, depressed mood, or hyperarousal are identified after a loss and are caused by the loss, they are part of "prolonged grief" or "complicated grief." These terms describe intense grief that lasts longer than would be expected according to social norms (for example, longer than one year) and can cause impairment in daily functioning [8]. For the purposes of this paper, the term "complicated grief" will be used, as it conveys that most grief reactions are normal, but some grief reactions can become pathological in duration, severity, and effect on daily function [9]. It is possible for complicated grief to be delayed in onset, last beyond the first year after a loss, and lead to enduring adjustment problems [10-12]. Complicated grief can also increase rates of cardiovascular disease, weight gain, diabetes, substance use, and suicide [13,14].

Underestimation of reproductive grief and disenfranchisement (meaning that typical societal recognition of grief is often not provided) of reproductive grief have resulted in a paucity of adequate screening options [15,16]. A short screening tool for complicated grief after a reproductive loss has never been developed [11,15]. The Edinburg Postpartum Depression Screening (EPDS), a widely utilized screening tool for perinatal depression, was not intended to detect complicated grief, and its wording may further disenfranchise and traumatize patients after loss. The EPDS is branded as a "postnatal" screening tool, which is an adjective many patients with reproductive loss do not feel matches their experience, especially after the termination of pregnancy, selective reduction, or first-trimester miscarriage. Many iterations of the EPDS also begin with the words "As you are pregnant or have recently had a baby...." However, the EPDS is increasingly administered early in pregnancy as well, which means the patient is administered the same survey before and after loss. This can act as a reminder or trigger, or it can project a message that the provider does not recognize the magnitude of the experience a patient has been through.

Despite this, the EPDS remains in practice for patients after losses due to a lack of alternative validated methods specific to these patients [17]. A 33-item scale, the Perinatal Grief Scale (PGS), exists to measure the intensity of perinatal grief in women and family members after a reproductive loss but is rarely used clinically due to its length [18].

The Brief Grief Questionnaire (BGQ) is a validated five-question screening tool useful in medical practices for identifying complicated grief and permitting prompt referral to appropriate mental health follow-up [19-21]. The BGQ was designed to screen adolescents or adults who are grieving a child, adolescent, or adult. It requires the insertion of a loved one’s name and refers to shared experiences and even photographs. The aim of this study was to modify the wording of the BGQ for reproductive loss and carry out a pilot validation study of the resulting new brief screening tool for medical providers to close a practice gap that was previously identified in clinical settings after reproductive loss [11]. This article was previously presented as a meeting abstract at the 43rd Annual Pregnancy Meeting for the Society for Maternal-Fetal Medicine on February 10, 2023.

## Materials and methods

The original creators of the BGQ were contacted and agreed that there were opportunities to improve its language for the target population. They agreed to allow the modification of the language of the BGQ to more appropriately target this population. To accomplish this, a team of one nurse, two physicians, and one patient advocate was assembled, all with significant experience with patients who experienced reproductive losses. Based on their extensive clinical experience, this multidisciplinary team adopted this instrument for reproductive losses, called the Reproductive Grief Screen (RGS; Table 1). Instrument development was carried out in an iterative process, with successive modifications of the BGQ being shown to clinical psychologists and patients. All modifications made to the BGQ are shown in Table 1, with explanations for each modification made.

**Table 1:** Comparison between the Brief Grief Questionnaire and the Reproductive Grief Screen, with differences in bold *The first column is a reproduction of the text of the Brief Grief Questionnaire (BGQ) as originally published by Ito et al. [21], with the permission of the authors. The remainder of the table reflects the original work.

Brief Grief Questionnaire^*^	Reproductive Grief Screen	Rationale for changes
How much are you having trouble accepting the death of ______________?	How much are you having trouble accepting your loss?	Embryos, fetuses, and even neonates may not have assigned names, so the name blank structure throughout the BGQ was removed. Depending on the type of experience, people who experience reproductive loss may have referred to this as only a pregnancy or an experience, not as a person.
How much does your grief still interfere with your life?	How much does your emotional response interfere with your life?	Complicated grief after reproductive loss is often labelled as another entity, such as hormonal changes, “baby blues,” perinatal depression, anxiety, or post-traumatic stress disorder. Based on clinical experience, we used “emotional response” to refer to the variety of possible symptoms after loss.
How much are you having images or thoughts of _____________ when s/he died or other thoughts about the death that really bother you?	How often are you having mental images of your loss or anything that reminds you of your loss?	The person who experienced a loss may have done so without being able to have specific images of the embryo or fetus, sometimes even without ultrasound images. This prompted generalizing “images or thoughts” to any reminder and adding the word “mental” to images to include imagery created by the patient’s imagination of a child or future adult.
Are there things you used to do when ______ was alive that you don’t feel comfortable doing anymore, or that you avoid? Like going somewhere you went with him/her, or doing things you used to enjoy together? Or avoiding looking at pictures or talking about _________? How much are you avoiding these things?	It is common for people who have experience a loss like yours to avoid doing things, being in situations, or being with people that remind them of their loss, such as attending baby showers, being around pregnant people, or having a baby. Some of these may apply to you and some may not. How often are you avoiding things you used to do before your loss?	Significant material was used to modify this item to normalize the variety of experiences people have after reproductive loss. Because of the nature of reproductive losses, the survey-taker and the embryo/fetus/neonate likely did not have time for significant shared life experiences together. Because of this, avoidance opportunities are typically situational (i.e., related to societal or personal experience with reproduction, such as baby showers). The lives of many young, reproductive-aged people are marked by child-rearing occasions for friends in the same age cohort so this is frequently an opportunity to observe avoidance. The content was removed about looking at pictures or doing things together, since these are usually not applicable to all types of reproductive loss.
How much are you feeling cut off or distant from other people since _________ died, even people you used to be close to like family or friends?	Since your loss, how much are you having difficulty connecting with other people, including family or friends?	This item was modified because removing the proper name made the sentence difficult to understand at the goal reading level for the final instrument.

For the purposes of this study, reproductive loss was defined to include miscarriage, stillbirth, neonatal death, infant death, selective reduction, or termination of pregnancy. The validation of the RGS received ethics approval from the University of Texas Health Science Center (HSC-MS-21-0213). One woman was recruited for reliability and discriminant validity testing. Participants were eligible if they were able to read English and had one or more reproductive losses between January 1, 2016, and December 30, 2021. Patients were recruited after low- and high-risk pregnancies at the University of Texas, as well as via social media. Significant efforts were made to protect the well-being of the participants. Partnerships with local providers in Houston were created to receive referrals for positive screens, especially regarding depressive symptoms. For participants who were not local to Houston, national hotlines were provided in cases of severe symptoms.

Participants were asked for some baseline variables to provide context to their replies, including age, race, ethnicity, history of conditions like generalized anxiety, PTSD, or depression prior to the pregnancy, gestational age at the time of loss (if prenatal), type of loss, time since loss, use of counseling and psychiatric medication during or after loss, and aspects of social support, such as whether the participant identified with a particular culture, set of beliefs, or religion and whether this was supportive during and after their loss. Three other validated perinatal grief assessment surveys were also administered to investigate the ability of the RGS to assess perinatal grief and depression (using the PGS), anxiety symptoms (the Panic Disorder Severity Scale, or PDSS), and trauma symptoms (the Impact of Events Scale, Revised, or IESR).

Categorical variables were analyzed with chi-square tests, normally distributed continuous variables were analyzed using t-tests, and non-normal continuous variables were analyzed using the Mann-Whitney U test. Descriptive statistics were calculated for each of the surveys. A Cronbach’s alpha coefficient was calculated to assess the validity of the RGS. A confirmatory factor analysis was then performed to assess the factorial validity of the RGS. Goodness-of-fit indices, including the root mean square error of approximation (RMSEA), comparative fit index (CFI), and standardized root mean squared residuals (SRMR), were calculated to assess unidimensional models. The average variance extracted (AVE) and composite reliability (CR) were calculated for discriminant validity. All p-values were two-tailed. Data were collected and managed in a secure, web-based software platform (Research Electronic Data Capture, or REDCap). R version 4.1.0 for Windows was used for analyses.

## Results

Of 140 women, 65 (46%) were recruited via social media, and 18 (13%) experienced their loss during a high-risk pregnancy. Baseline characteristics of the population are shown in Table 2. Most participants were white and non-Hispanic, and most experienced a loss before delivery. The mean time since the loss was two years (IQR 1-5 years).

**Table 2:** Demographics for 140 women Data are displayed as median [IQR] or number (percent).

Variable	N = 140
Age (years)	35.0 [31.2–39.0]
Race	
White	102 (75)
Black	9 (7)
Asian	8 (6)
More than one race	8 (6)
Other	10 (7)
Years since loss	2.0 [1.0–5.0]
Timing of loss	
Before delivery	113 (81)
After delivery	26 (19)
Weeks of pregnancy (if pregnant)	11.0 [7.0–21.0]
Loss associated with a high-risk pregnancy	18 (13)
Lost more than one fetus/neonate during the loss	25 (18)
Experienced more than one loss	74 (53)
Number of losses (if multiple)	2.0 [2.0–3.0]
Used psychiatric medications during recovery	104 (74)
Had a subsequent pregnancy	58 (42)
Identify as having a religion	117 (84)
Identify with a particular culture/tradition	48 (34)

**Figure 1:**
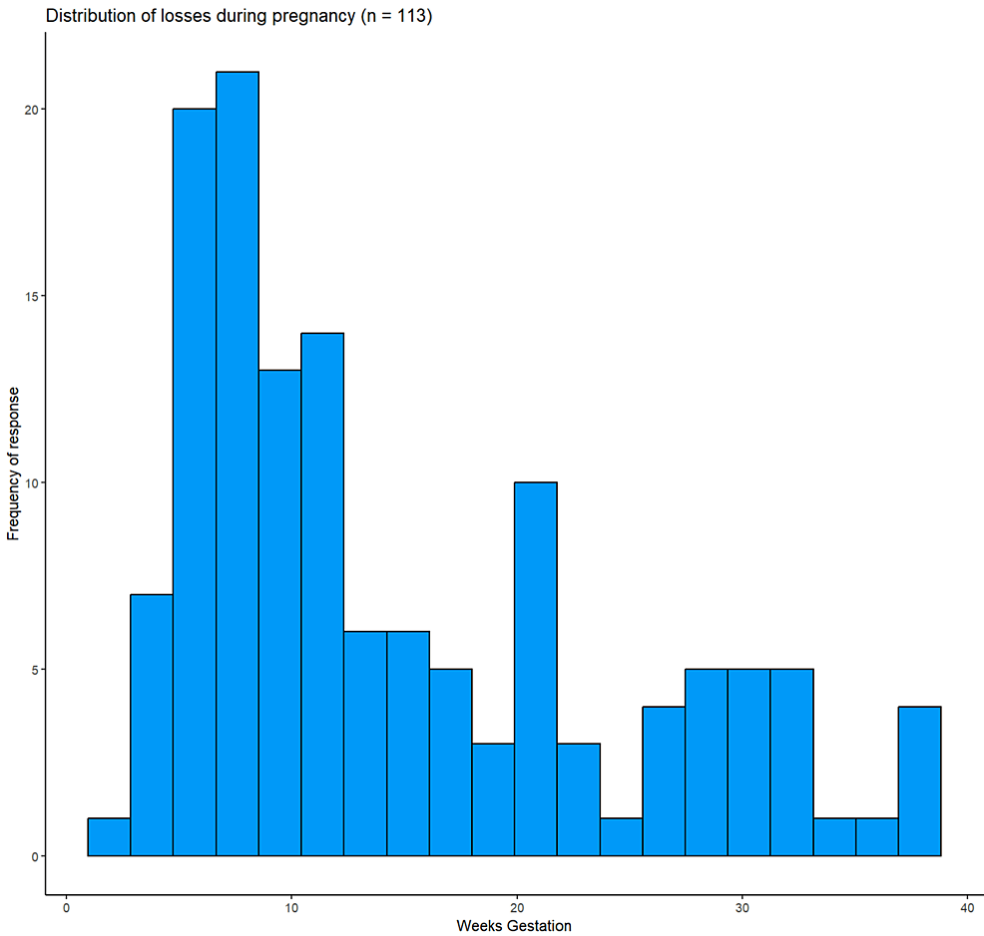
Distribution of timing in 113 losses which occurred during pregnancy Postnatal losses are not depicted.

Seven women reported that one or more losses were terminations of pregnancy with one selective reduction. Three women reported at least one loss being a stillbirth (fetal death after 20 weeks). Four women reported neonatal or infant loss after birth. The remainder experienced one or more deaths of a fetus or embryo prior to 20 weeks. For losses prior to delivery, the mean gestational age at loss was 14 weeks and 4 days (IQR 7.1-21.7 weeks; Figure 1).

**Figure 2:**
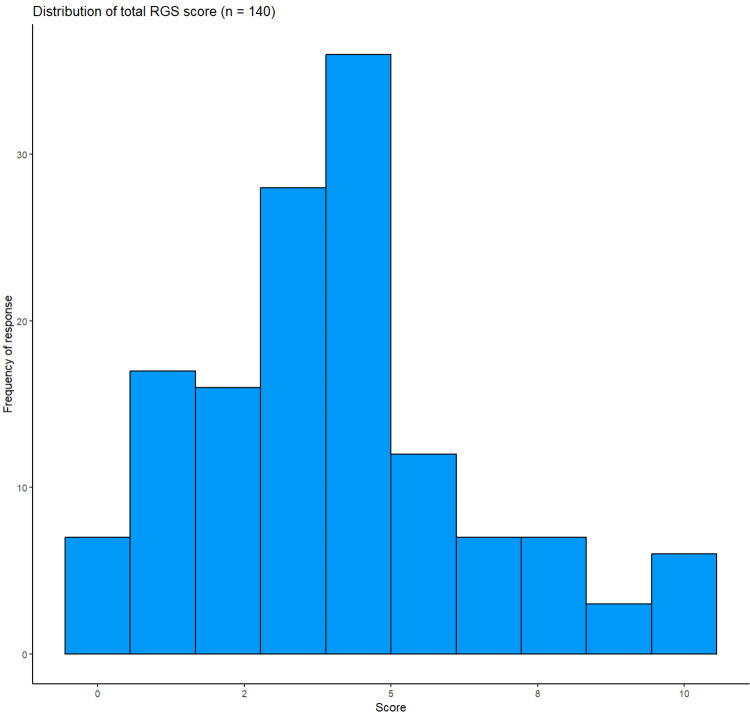
Distribution of total Reproductive Grief Screen score (n = 140)

The mean RGS score was 4.02 (IQR 2.27-5.77), the skewness was 0.58, and the kurtosis was 2.74. The distribution of scores is shown in Figure 2, and item-level data are shown in Table 3. Cronbach's alpha was 0.77 (95% confidence interval, CI, was 0.69-0.83).

**Table 3:** Item characteristics SD: standard deviation. Response values were coded as 0 (“Not at all”), 1 (“Sometimes”), or 2 (“A lot”). Item-total correlation tests were performed; item-total correlations to assess whether an individual question ("item") deviates from the behavior of the entire set of items, and could thus be discarded as an unhelpful question to ask patients.

Item	Mean ± SD	Item-Total Correlation
How much are you having trouble accepting your loss?	0.76 ± 0.71	0.55
How much does your emotional response interfere with your life?	0.79 ± 0.64	0.53
How often are you having mental images of your loss or anything that reminds you of your loss?	1.08 ± 0.61	0.52
It is common for people who have experience a loss like yours to avoid doing things, being in situations, or being with people that remind them of their loss, such as attending baby showers, being around pregnant people, or having a baby. Some of these may apply to you and some may not. How often are you avoiding things you used to do before your loss?	0.76 ± 0.79	0.54
Since your loss, how much are you having difficulty connecting with other people, including family or friends?	0.64 ± 0.73	0.61

RGS scores and item means did not differ by race, a supportive religion or culture, or losing more than one embryo or fetus. Having a loss after delivery was strongly associated with more reminder symptoms (p = 0.017) and a higher RGS overall (p = 0.003). More reminder symptoms were also associated with the use of psychiatric medications (p = 0.009). A subsequent pregnancy was associated with a lower overall RGS score (p < 0.001) and a lower score on four of five individual items [interference (p < 0.001), reminders (p = 0.004), avoidance (p = 0.001), and connecting (p = 0.006)]. Suboptimal timing of the next pregnancy, as judged by the patient, was associated with higher total RGS scores (p = 0.017) and more reminder symptoms (p = 0.002).

**Figure 3:**
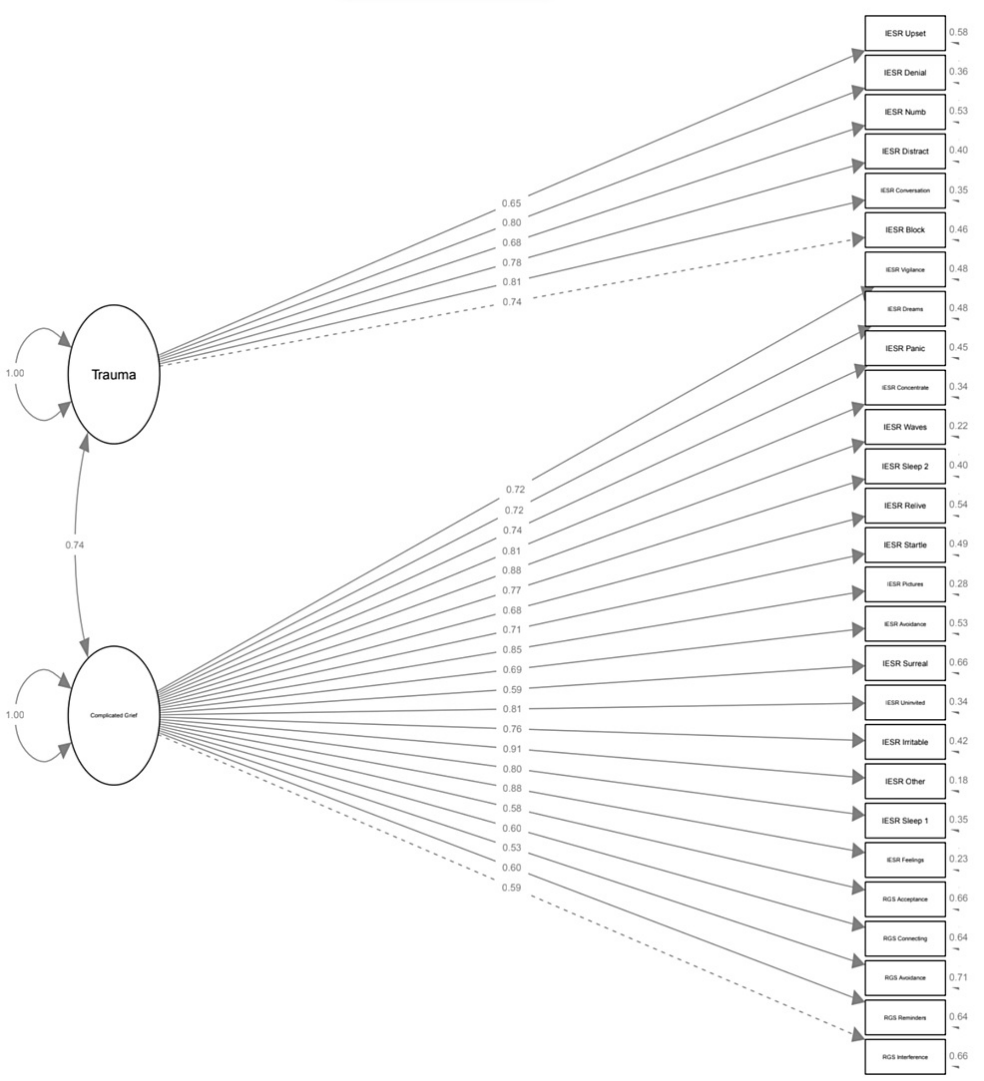
Crossover between IESR and RGS items All items labeled “IESR” belong to a trauma scale but cross-load onto the Complicated Grief Factor (lower left) with items labeled “RGS,” which correctly load. Ideally, the items (questions) in the trauma survey (IESR) would "load" onto the trauma factor, and the items in the reproductive grief screen (RGS) would load onto the complicated grief factor. This would indicate that the two surveys measure different symptom pools. However, multiple IESR items "cross-load" (load onto another factor) onto the complicated grief factor. The cross-loading items relate to vigilance, dreams, panic symptoms, concentration, symptoms coming in waves, effects on sleep, reliving experiences, being easily startled, avoidance, and irritability. This suggests that these symptoms in particular may overlap with complicated grief, or that the RGS may be overly sensitive for trauma symptoms.

Nine eigenvalues of the exploratory factor analysis for all items, including RGS, PDS, and IESR, exceeded 1 (range, 1.05-25.9). All of the RGS items loaded onto the complicated grief factor (factor loadings of 0.51-0.85), and none cross-loaded onto the anxiety or trauma factors. PDSS items loaded onto the anxiety factor (range 0.82-0.94) without cross-loading, but multiple IESR items cross-loaded onto the complicated grief factor (Figure 3).

A confirmatory factor analysis was done between RGS and PGS items. The goodness of fit indices of the model meet the Fornell-Larcker criterion (RMSEA = 0.167, CFI = 0.89, SRMR = 0.06). The Fornell-Larcker criterion is a test for discriminant validity in investigator-designed survey instruments [22]. The AVE for RGS and PGIS were 0.42 and 0.46, respectively, with CRs of 0.78 and 0.93, respectively. The correlation between the latent factors of RGS and PGS was −0.95, suggesting convergence and measurement of similar symptoms. The correlations between the latent factor of RGS and PDSS and between RGS and coping variables were low (0.66 and −0.39, respectively), suggesting RGS did not simply assess non-specific anxiety or inadequate support.

## Discussion

A five-question survey for complicated grief after the reproductive loss was developed by experts and tested in 140 women, with mixed results. Cronbach’s alpha was acceptable (>0.7), but item-total correlations were low (<0.67), suggesting reliability can be improved. The survey meets the Fornell-Larcker criterion, which is an acceptable first achievement for an investigator-designed survey [22]. A high correlation between RGS and PGS suggests measurement of the same perinatal grief that this previously validated, longer instrument assesses. Cross-loading with IESR items and the overall correlation between RGS and IESR latent variables (0.92) suggest that symptoms of complicated grief overlap with trauma symptoms. This is consistent with previous literature, which shows that almost one-third of patients meet diagnostic criteria for post-traumatic stress disorder one month after the reproductive loss, dropping to 18% nine months after the loss - this is higher than the rates of anxiety (24% and 17%, respectively) or depression (11% and 6%) after reproductive loss [23].

The majority of participants (51%) had a score of 4 or more, which in the original BGQ is a positive screening test [21]. This proportion is greater than the reported incidence of complicated grief in the literature (15-25%), which may indicate an overly sensitive test or describe a previously unrealized degree of symptoms, as missing grief reactions are common in practice [24].

Weaknesses of this study include its low item-total correlations at this early stage as well as its non-diverse demographic representation. The sample under-represents minority populations, but it accurately reflects the distribution of reproductive losses as mostly occurring in early pregnancy. Another limitation of this study is that depressive symptoms were not a large focus of the various instruments, except for parts of the PGS. Complicated grief and comorbid postnatal depression may not have been teased apart adequately by this study. Strengths include the breadth of statistical probing into the performance of this investigator-designed survey.

Prior to the RGS seeing clinical use, it must be modified to achieve higher reliability, its acceptability must be tested among people who have experienced various types of reproductive loss, and the sensitivity and specificity of the RGS must be determined. These three aspects of the study are necessary but separate in scope from the work documented in the current report, which aimed only to see whether an existing survey could be modified and retain basic validity statistics. The immediate next step is a mixed-methods investigation of the wording of the instrument to continue to optimize its validity and test its acceptability, and this is ongoing. Sensitivity and specificity testing for the appropriate cutoff of the RGS (which for the BGQ is 4) requires that participants complete the RGS and undergo a detailed interview with a psychiatrist to establish or rule out the diagnosis of complicated grief, which is a step further in the future.

## Conclusions

No short, clinical tool exists to detect complicated grief after reproductive loss; while a specific tool exists, it asks 33 numbers and words questions that personify the reproductive loss, which is not felt by all persons who have experienced these life events. This study designed and validated a five-item survey to screen for complicated grief, using input from a multidisciplinary team of medical providers and advocates.

At this stage, the clinical implications of this preliminary work are few, but clinicians should be vigilant for mental health symptoms that may affect daily function in people who experience reproductive loss. The next steps include optimizing this tool, determining its sensitivity and specificity for the detection of complicated grief as opposed to post-traumatic stress, anxiety, or postnatal depression, and determining how the RGS should be scored clinically. In the future, an optimized brief tool such as the one created in this study may be a way to screen patients after reproductive loss.
